# Epigenetic ageing is distinct from senescence-mediated ageing and is not prevented by telomerase expression

**DOI:** 10.18632/aging.101588

**Published:** 2018-10-17

**Authors:** Sylwia Kabacik, Steve Horvath, Howard Cohen, Kenneth Raj

**Affiliations:** 1 Cellular Biology Group, Radiation Effects Department, Centre for Radiation, Chemicals and Environmental Hazards (CRCE) Public Health England (PHE) Dicot, Chilton OX11 0RQ, Oxfordshire, United Kingdom; 2 Departments of Human Genetics and Biostatistics, David Geffen School of Medicine, University of California Los Angeles, Los Angeles, CA 90095, USA; 3 Elizabeth House Medical Practice, Warlingham, Surrey CR6 9LF, United Kingdom

**Keywords:** epigenetic ageing, hTERT, epigenetic clock, ageing, senescence

## Abstract

The paramount role of senescent cells in ageing has prompted suggestions that re-expression of telomerase may prevent ageing; a proposition that is predicated on the assumption that senescent cells are the sole cause of ageing. Recently, several DNA methylation-based age estimators (epigenetic clocks) have been developed and they revealed that increased epigenetic age is associated with a host of age-related conditions, and is predictive of lifespan. Employing these clocks to measure epigenetic age *in vitro*, we interrogated the relationship between epigenetic ageing and telomerase activity. Although hTERT did not induce any perceptible change to the rate of epigenetic ageing, hTERT-expressing cells, which bypassed senescence, continued to age epigenetically. Employment of hTERT mutants revealed that neither telomere synthesis nor immortalisation is necessary for the continued increase in epigenetic age by these cells. Instead, the extension of their lifespan is sufficient to support continued epigenetic ageing of the cell. These characteristics, observed in cells from numerous donors and cell types, reveal epigenetic ageing to be distinct from replicative senescence. Hence, while re-activation of hTERT may stave off physical manifestation of ageing through avoidance of replicative senescence, it would have little impact on epigenetic ageing which continues in spite of telomerase activity.

## Introduction

Although ageing is readily observed at the level of the organism, our understanding of why and how this process occurs has remained speculative until normal human cells were successfully cultured outside the body, where they were found to have a finite capacity to proliferate.

Hayflick estimated that a population of human cells grown *ex vivo* can double approximately sixty times after which they adopt a permanent state of dormancy termed replicative senescence [1,2]. The cause of this natural limitation to proliferation was eventually found to lie in the “end-replication problem”, which if not addressed by the cell, would lead to telomere attrition at every round of DNA replication [3,4]. It was eventually demonstrated that this does indeed occur and when telomeres shorten to a critical length they trigger cells to adopt the senescent state [5,6]. The identification of telomerase, which replicates telomeres [7,8], and the fact that most adult somatic cells do not produce this enzyme, provided the last major piece of the puzzle that describes the ageing process from events beginning with molecules, proceeding to cells and culminating in the organism. Significantly, this chain of events can be prevented by ectopic expression of hTERT, the catalytic sub-unit of telomerase, which can preserve telomere length and avert senescence of some cells [9,10]. Impressively, these profound insights into the process of human ageing were acquired from careful study of *ex vivo* cell behaviour.

It was initially thought that the functional and physical deterioration that characterise organismal ageing are a result of insufficient replenishment of cells due to telomere-mediated restriction of cellular proliferation. Senescent cells, which accumulate increasingly in tissues in function of age, were assumed to be passive and merely a consequence of the above-described processes. This notion was short-lived when senescent cells were found to secrete molecules that are detrimental to cells and tissues; a cellular characteristic described as senescence-associated secretory phenotype (SASP) [11–13]. The role of senescent cells in actively causing age-related physical deterioration was elegantly revealed when reversal of ageing phenotype in organs and tissues was observed following the removal of senescent cells in mice [14]. As such, it would follow that if cells were prevented from becoming senescent in the first place, ageing could be avoided. Although there are external instigators such as stress and DNA damage that can also cause cells to become senescent [15], replicative senescence is particular in that it is an intrinsic feature that is part of cellular proliferation and occurs even in an ideal environment. As expression of hTERT has been repeatedly demonstrated to prevent replicative senescence of many different cell types, it is reasonable to consider ectopic expression or re-activation of endogenous hTERT expression as potential means to prevent replicative senescence, delay ageing and improve health [16].

The above proposition would be valid if senescent cells were indeed the only cause of ageing. Relatively recently, an apparently distinct form of ageing, called epigenetic ageing was described (reviewed in [17]). This discovery stems from observations that the methylation states of some specific cytosines that precede guanines (CpGs) in the human genome changed rather reliably and strictly with age [18–22]. This allowed supervised machine learning methods to be applied to DNA methylation data to generate DNA methylation-based age estimators (epigenetic clocks) of epigenetic age, which in the majority of the human population is similar with chronological age [23–27]. The difference between epigenetic age and chronological age, which reflects the rate of epigenetic aging, carries biological significance: increased epigenetic aging is associated with numerous age-related pathologies and conditions [17,28–41]. Conversely, healthy lifestyle and diet is associated with younger epigenetic age [17,42]. Furthermore, epigenetic age can be reversed or reset, as expression of Yamanaka factors in somatic adult cells reset their epigenetic ages to zero [26,43]. Hence, epigenetic age is not merely an alternative means of determining chronological age but is to some degree a measure of biological age or health; a proposition that is further supported by the impressive demonstration that acceleration of epigenetic ageing is associated with increased risk of all-cause mortality [34,39]. Collectively, the descriptions above highlight the fact that epigenetic ageing, in spite of the mathematical origins of its discovery, is not a mathematical contrivance but a genuine ageing process innate in cells.

Several DNAm-based biomarkers have been reported in the literature that differ in terms of their applicability (some were developed for specific tissues such as blood) and their biological interpretation. The pan-tissue epigenetic clock developed by Horvath [26] is applicable to almost all sources of DNA with the exception of sperm. The resulting age estimate by this clock is referred to as epigenetic age or more precisely DNAm age. Although the pan-tissue epigenetic clock is highly accurate and applicable to the vast majority of tissues in the body, it performs sub-optimally when estimating the age of fibroblasts. In response to this, we recently developed a new epigenetic age estimator, referred to as skin & blood clock that is more accurate in estimating age of different cell types including fibroblasts, keratinocytes, buccal cells, blood cells, saliva and endothelial cells [44]. Studies employing skin & blood clock and the pan-tissue epigenetic age clock revealed a startling consistency of epigenetic age across diverse tissues from the same individual, even though cellular proliferation rates and frequencies of these tissues are not the same [26,44]. This suggests that the ticking of the epigenetic clock is not a reflection of proliferation frequency, which is in stark contrast to telomere length, which enumerates cellular division. It would therefore appear that the process of epigenetic ageing is distinct from that which is driven by telomere-mediated senescence. To understand their relationship or interaction, if one indeed exists, we set out to test the impact of hTERT on epigenetic ageing. To this end we employed wild type hTERT that can prevent telomere attrition and its mutants that cannot [45], with some still able to nevertheless prolong cellular lifespan [46]. Expressing these hTERT constructs in primary cells from numerous donors, ages and cell types, we observe that while hTERT expression can indeed prevent cellular senescence, it does not prevent cells from undergoing epigenetic ageing and that extension of cellular lifespan is sufficient to support continued epigenetic ageing of the cell. These simple observations provide a very important piece to the puzzle of the ageing process because it reveals the distinctiveness of epigenetic ageing from replicative senescence-mediated ageing. They provide further empirical support to the epidemiological observation that hTERT variant that is associated with longer telomeres are also associated with greater epigenetic ageing [47].

## RESULTS

**Figure 1:**
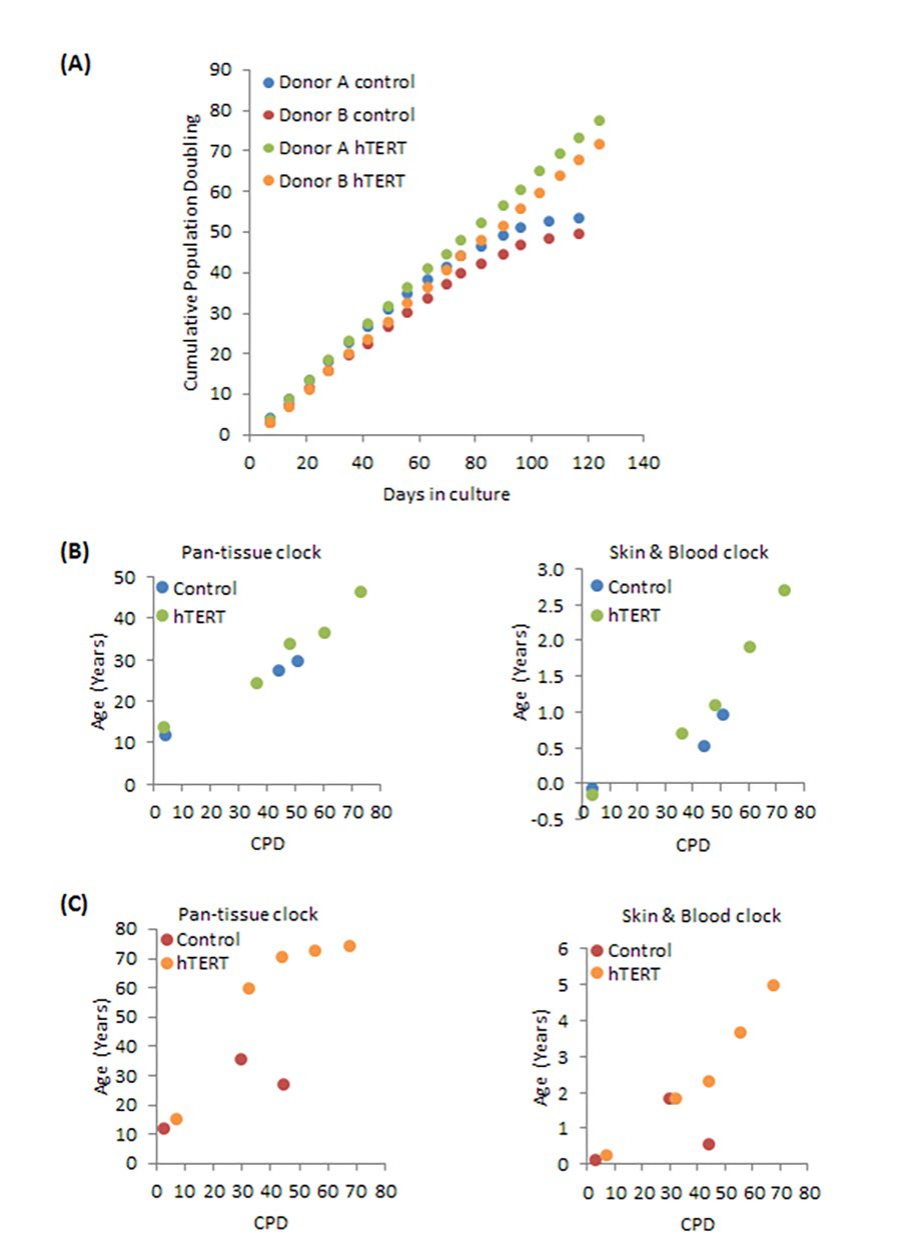
**Effects of hTERT on growth and epigenetic ageing of human primary neonatal fibroblasts.** (**A**) Growth dynamics of primary cells from two different donors (A and B) transduced with either empty vector (control) or hTERT expressing vector (hTERT). The ages of a selection of cell passages of donor A (**B**) and donor B (**C**) were imputed by the pan-tissue clock (left panel) or the skin & blood clock (right panel). The ages are plotted against cumulative population doubling (CPD) that corresponded to the passage of cells that were analysed.

To test the effect of hTERT on epigenetic ageing, we first transduced primary neonatal foreskin fibroblasts with hTERT vectors and subjected them and the control isogenic cells, which harbour empty vectors, to continuous culture with passaging. The growth curve in Figure 1A shows that control fibroblasts from neonatal donors A and B (blue and red dots) senesced after about a hundred days in culture and having doubled approximately 50 times (Supplementary Figure 1 and 2A). Unsurprisingly, cells bearing hTERT expression vector bypassed replicative senescence. They proliferated unabated beyond 130 days and 75 cumulative population doubling. The last green and orange dots represent the time-point at which the experiments were terminated, and not the end of cellular viability. These cells have effectively become immortalised. Cellular DNA from a selection of cell passages were subjected to methylation analyses with Illumina EPIC array. The resulting data were processed using the pan-tissue clock and the new skin & blood clock. The results in Figure 1B (for Donor A) and Figure 1C (for Donor B) show control cells to age in culture and this was not perturbed by hTERT expression. Importantly, cells transduced with hTERT not only evaded replicative senescence, their epigenetic ages continued to steadily increase past the point of replicative senescence encountered by their respective isogenic control counterparts (last blue and red dots). While this behaviour is observed with results derived from both epigenetic ageing clocks, the pan-tissue clock clearly displays an off-set from the chronological age of the neonatal cells, which is zero years, as correctly indicated by the skin & blood clock. Incidentally, this systematic offset/error in accurately estimating the epigenetic age of young fibroblasts was one of the reasons for developing the skin & blood clock.

**Figure 2:**
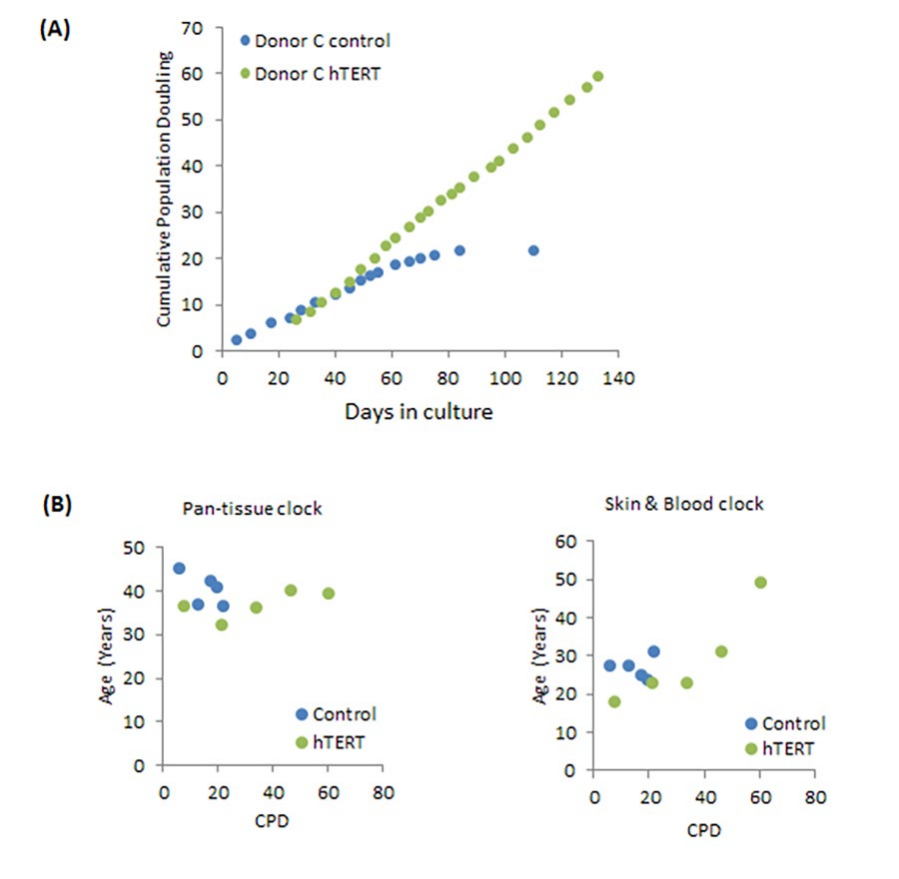
**Effects of hTERT on growth and epigenetic ageing of adult primary human coronary artery endothelial cells.** (**A**) Growth dynamics of primary cells from one donor (C) transduced with either empty vector (control) or hTERT expression vector (hTERT). (**B**) The ages of a selection of cell passages of donor C were imputed by the pan-tissue clock (left panel) or the skin & blood clock (right panel). The ages are plotted against cumulative population doubling (CPD) that corresponded to the passage of cells that were analysed.

To ascertain whether the effect of hTERT seen in neonatal foreskin fibroblasts was observable in cells from another tissue and age, we utilised human coronary artery endothelial cells (HCAEC) from adult donor (Donor C; 19 years old). The growth dynamics of these cells as shown in Figure 2A are similar in principle with those of the neonatal fibroblasts, with the difference being the earlier time-point at which the control cells senesce (Supplementary Figure 2B). This is consistent with them being adult cells and as such would have lower replicative potential. As with neonatal fibroblasts, the adult HCAEC expressing hTERT were also immortalised. A startling difference however, is apparent when the ages of these cells were estimated by the two clocks (Figure 2B). While once again the skin & blood clock showed hTERT-expressing cells, which bypassed replicative senescence, to continue ageing steadily, the epigenetic age estimates from the pan tissue clock were much higher and with no significant change in age (Figure 2B). We have observed similar pattern with HCAEC isolated from another donor (26 years old) [44].

**Figure 3:**
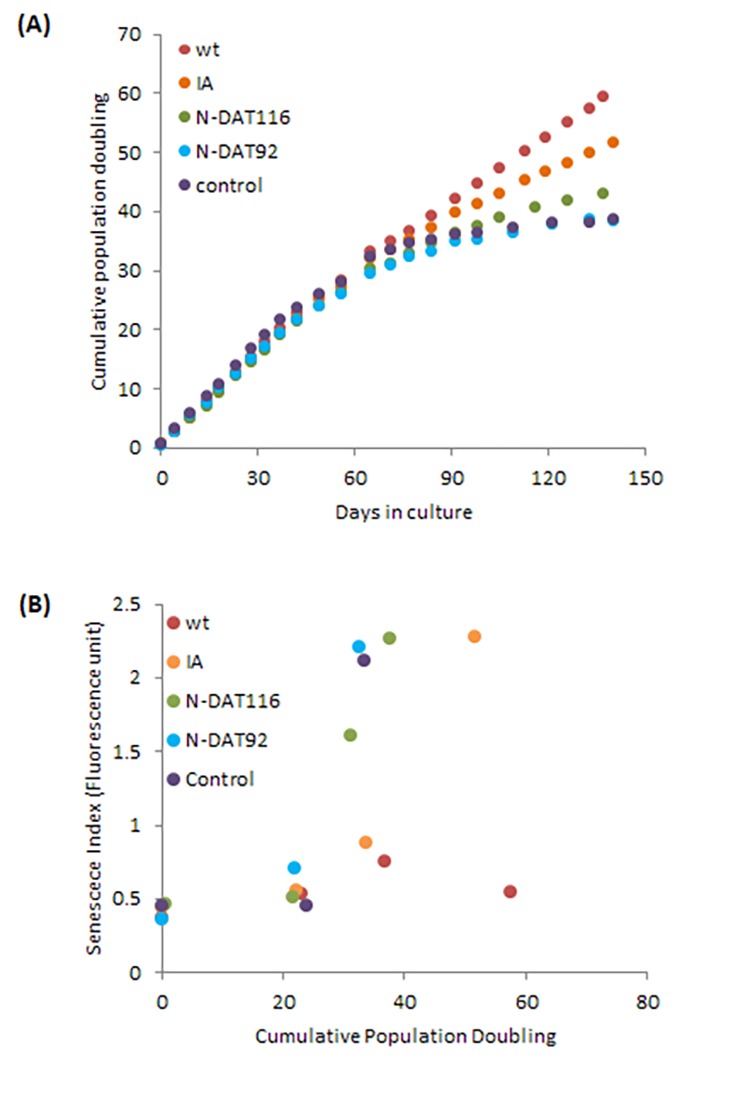
**Effects of hTERT and its mutants on growth and senescence of human primary neonatal fibroblasts.** (**A**) Growth dynamics of primary cells transduced with either empty vector (control), vector expressing wildtype hTERT (WT), IA mutant (IA), N-DAT116 mutant (N116) or N-DAT92 mutant (N92). (**B**) Cells from a selection of passages were subjected to senescence assay and the senescence index is plotted against cumulative population doubling that corresponded to the passage of cells that were analysed.

**Figure 4:**
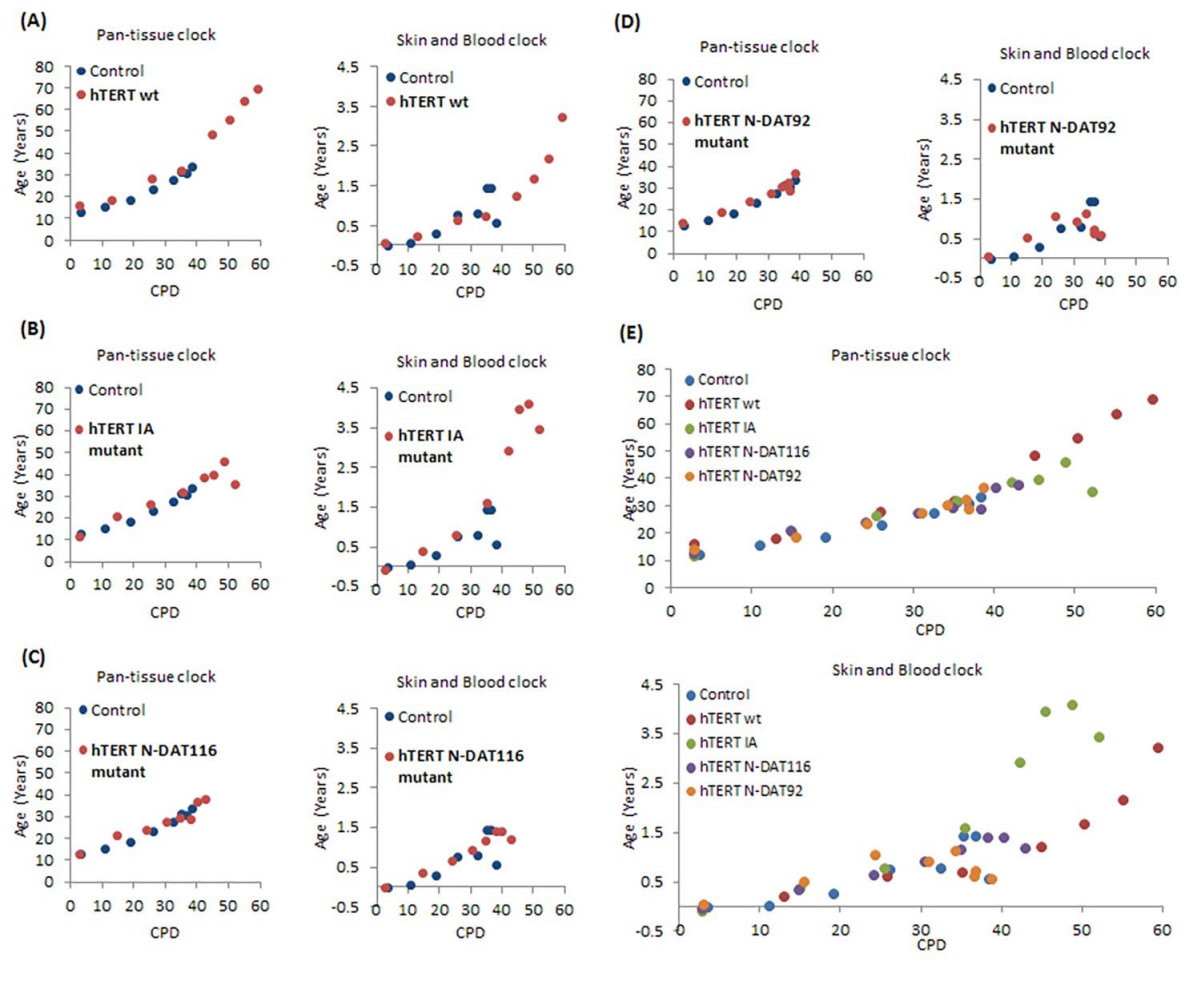
**Effects of hTERT and its mutants on epigenetic ageing of human primary neonatal fibroblasts.** Ages of primary human neonatal fibroblasts bearing empty vector (blue dots), wildtype hTERT (**A**), IA mutant (**B**), N-DAT116 mutant (**C**) or N-DAT92 mutant (**D**) were determined using the pan-tissue clock (left panel) and the skin & blood clock (right panel). (**E**) Composite plot of all the hTERT mutants. The ages of cells from a selection of passages are plotted against cumulative population doubling (CPD) that corresponded to the passages of cells that were analysed.

To further investigate the relationship between hTERT and epigenetic ageing, we employed a previously validated and published panel of hTERT mutants which all possess catalytic activity but are compromised in one or several hTERT properties, namely, extension of replicative lifespan, telomere synthesis or immortalisation (Table 1 and Supplementary Figure 3) [48]. The growth characteristics of the neonatal foreskin fibroblasts transduced with these vectors confirmed that cells expressing wildtype hTERT bypassed replicative senescence (Figure 3) and aged steadily pass the point of replicative senescence encountered by the control cells (Figure 4A). Notably the hTERT IA mutant [46], which can significantly extend replicative lifespan (Figure 3) but can neither replicate telomeres nor immortalise cells, is also able to elicit steady epigenetic ageing pass the point of replicative senescence of the control cells (Figure 4B). This feature is particularly important because it shows that neither telomere synthesis nor immortalisation contribute to the steady rise in epigenetic ageing seen with hTERT-expressing cells. Instead extension of cellular lifespan appears to be the critical property associated with increased epigenetic ageing. Accordingly, the N-DAT116 mutant [46], which was reportedly also able to extend cellular lifespan of human mammary epithelial cells [46,48], but did so only very marginally with neonatal fibroblasts, did not cause a substantial rise in epigenetic ageing (Figure 4C). Likewise the N-DAT92 [46] hTERT mutant that does not increase lifespan also did not increase epigenetic ageing (Figure 4D). The patterns described above largely hold true between the two epigenetic age clocks. It is evident that age scatter plots derived from the pan-tissue clock appear more linear, as is seen in the composite plot in Figure 4E. This is not surprising as the spread of ages estimated by it is much greater than those by the skin & blood clock. Notwithstanding the age off-set that is apparent with the pan-tissue clock, and the greater noise of the skin & blood clock, their results are consistent in showing that while hTERT can prevent replicative senescence, it is ineffective in stopping epigenetic ageing.

**Table 1:** Mutants of hTERT used in the experiments.

	**Catalytic activity**	**Extension of Life-span**	**Telomere Synthesis**	**Immortalisation**
**hTERT wt**	**YES**	**YES**	**YES**	**YES**
**hTERT IA**	**YES**	**YES**	**NO**	**NO**
**hTERT N-DAT116**	**YES**	**YES**	**NO**	**NO**
**hTERT N-DAT92**	**YES**	**NO**	**NO**	**NO**

The characteristics of the hTERT mutants in the left column are indicated by yes or no in regards to whether they are capable of lifespan extension, telomere synthesis or cellular immortalisation.

## DISCUSSION

We carried out these simple but very long-drawn out experiments to interrogate the connection, if there was one, between replicative senescence (as mediated by telomeres) and epigenetic ageing. In order to interpret these findings correctly, it is necessary to be reminded that epigenetic age, as imputed by the epigenetic clocks is neither a measure of cellular proliferation rate nor a measure of proliferation or passage number. This is evident from the fact that epigenetic age of isogenic tissues (from the same individual) with high and low turn-over rates (blood and heart for example) are similar [26,44]. Epigenetic ageing is also not a measure of senescent cells, as is evident from this study where epigenetic age continues to rise inexorably in hTERT-expressing cells, which do not senesce. These characteristics underline the distinctiveness of epigenetic ageing from replicative senescence-mediated ageing, which supports our previous findings [49] and three genome-wide association studies (GWAS) which did not detect a relationship between telomere length and epigenetic ageing [50–53].

Do these two different ageing processes interact? Since hTERT-expressing cells (subsequently referred to as hTERT cells) exhibit greater age, it would appear that hTERT promotes epigenetic ageing. This however is not the case because hTERT cells do not exhibit higher ages than control cells prior to the point of replicative senescence of the latter. This is evident from the similar gradient of age increase between control and hTERT cells. The acquisition of greater age by hTERT cells after senescence of the control cells is a smooth continuum of the ageing gradient. As such, observations from these experiments do not support the proposition that hTERT stimulates epigenetic ageing. Instead, by causing cells to bypass replicative senescence, hTERT allows the inherent process of epigenetic ageing, which occurs regardless of its presence, to continue. Put simply, while hTERT may appear on the surface, to exacerbate the epigenetic ageing of cells, in truth hTERT, by preventing telomere attrition, prolonged the lifespan of the cells, allowing them growing older (as measured by the epigenetic clocks).

Accordingly, telomere synthesis and immortalisation are not necessary for the acquisition of greater age; a point that is clearly made by hTERT IA mutant, which increased epigenetic age in spite of its inability to maintain telomere length or immortalise cells, but is still able to extend lifespan [46]. It is interesting that although both IA and N-DAT116 mutants are reportedly able to increase life-span of human mammary epithelial cells [46,48], the magnitude of their effect in human neonatal fibroblasts is very different. The hTERT IA mutant, which is far more effective in this regard, is also highly effective in increasing epigenetic age. The hTERT N-DAT116 mutant on the other hand induces only a marginal increase in lifespan and accordingly, no age increase beyond the control cells is evident (measured by the skin and blood clock) and a correspondingly small increase as measured by the pan-tissue clock. These observations are consistent and they point to increased epigenetic ageing in hTERT cells as a result of extension of lifespan.

This conclusion is also consistent with the recently reported genome-wide association study (GWAS) that identified an variant of hTERT that is associated with increased epigenetic ageing [47]. Interestingly, this allele is also associated with longer telomeres. This observation appeared counter-intuitive in the first instance because short telomeres are unequivocally associated with greater age. As such hTERT variants that generate short telomeres would be expected to be associated with increased epigenetic ageing. The apparent paradox disappears when it is realised that while telomere length undoubtedly records the proliferative history of the cell, it also indicates its proliferative or lifespan potential. As such, cells with longer telomeres have longer lifespan, and as empirically demonstrated here, longer lifespan is accompanied by greater epigenetic ageing. In other words, ectopic expression of hTERT (in this study) and expression of a natural hTERT locus variant associated with longer telomeres *in vivo* (suggested by GWAS) would increase cellular lifespan, with the consequence of greater epigenetic ageing.

The distinctiveness and independence of epigenetic ageing from replicative senescence, exerts a serious impact on ageing intervention strategies. It is likely that re-activation of hTERT expression or elimination of senescent cells will go some way to mitigate the effects of ageing. These measures however, are unlikely to be sufficient to halt ageing altogether since they will not prevent epigenetic ageing. Interventions that prevent or eliminate senescent cells hold great promise for extending human healthspan. Our study suggests that these interventions might not arrest epigenetic aging, which is disconcerting considering the over-whelming evidence that point to the association between accelerated epigenetic ageing and a host of disparate diseases and conditions [17,28–41]. To what extent epigenetic aging of various cells causes the decline in organ function remains an area of active research, but it is arguable that to maximise healthspan there may be a need to develop compounds that target epigenetic ageing as well. In this regard the new skin & blood clock can form the basis of an assay to test for such interventions. This clock out-performs the pan-tissue clock which is already highly accurate for most tissues and cells in the body, but for unknown reasons exhibit a considerable age off-set when used on some cells cultured *in vitro*. Furthermore, the pan tissue clock also differed substantially from the skin & blood clock when applied to adult human coronary artery cells: unlike the skin & blood clock, it led to a substantial offset and did not reveal the increase of epigenetic age in function of cell growth. We have at present, no explanation for this curious observation. Overall, it appears that the skin & blood clock is more suitable for cultured cells, which is particularly important because the ability to accurately measure cellular age *in vitro* will allow the yet unknown mechanism of the epigenetic clock to be elucidated more easily. Gratifyingly, the compatibility of the skin & blood clock with cells *in vitro* does not come with any loss of compatibility with cells *in vivo,* as was recently described [44].

In summary, these experiments greatly advance our understanding of the connection between epigenetic ageing and senescence-mediated ageing and on this, they have successfully provided empirical evidence that these two mechanisms of ageing are distinct and uncoupled. With the tools that are available (epigenetic clock and primary cells) and the realisation of the difference between these two ageing processes, we are in much improved position to proceed towards understanding the mechanism of epigenetic ageing and its role in human pathology.

## MATERIALS AND METHODS

### Isolation and culture of primary cells

Primary human neonatal fibroblasts were isolated from circumcised foreskins. Informed consent was obtained prior to collection of human skin samples with approval from the Oxford Research Ethics Committee; reference 10/H0605/1. The tissue was cut into small pieces and digested overnight at 4 °C with 0.5 mg/ml Liberase DH in CnT-07 keratinocyte medium (CellnTech) supplemented with penicillin/streptomycin (Sigma) and gentamycin/amphotericin (Life Tech). Following digestion, the epidermis was peeled off from the tissue pieces. The de-epidermised tissue pieces were placed faced down on plastic cell culture plates and allowed partially dry before addition of DMEM supplemented with 10% FBS and antibiotics. After several days incubation in a 37 °C, 5% CO_2_ humidified environment, fibroblasts can be seen to migrate out from the tissue pieces and when their growth reached confluence, they were trypsinised, counted and seeded into fresh plates for experiments. Adult human coronary artery endothelial cells (HCAEC) were purchased from Cell Applications (USA) and cultured in MesoEndo Cell Growth Medium (Sigma) at 37 °C humidified incubator with 5% CO_2_.

### Neonatal foreskin fibroblasts

100,000 cells were seeded into a 10cm plate and cultured as described above. Upon confluence the cells were harvested with trypsin digestion followed by neutralisation with soybean trypsin inhibitor. The number of cells was ascertained and 100,000 was taken and seeded into a fresh plate. The remaining cells were used for DNA extraction. Population doubling was calculated with the following formula: [log(number of cells harvested) – log(number of cells seeded)] x 3.32. Cumulative population doubling was obtained by addition of population doubling of each passage.

### Adult human coronary artery endothelial cells

500,000 cells were seeded into a fibronectin-coated 75cm^2^ flask and cultured as described above. The procedure of passing the cells, counting and ascertaining population doubling is similar to those described for neonatal foreskin fibroblasts above.

### Retroviral-mediated transduction of cells with hTERT vectors

The procedure of transducing neonatal foreskin fibroblasts and adult human coronary artery endothelial cells are similar with the exception of the media that is used to collect the retroviruses.

The various retroviral vectors bearing wildtype hTERT (Addgene, cat. 1774), IA mutant, N-DAT116 mutant, HA mutant or N-DAT92 mutant (kind gifts from Dr. Christopher Counter, USA) were individually transfected into Phoenix A cells using the calcium chloride method according to the manufacturer’s instructions (Profection Cat No: E1200 Promega). They next day, media were removed from the transfectants and replaced with either DMEM supplemented with 10% foetal calf serum (for subsequent use on neonatal fibroblasts) or MesoEndo media (for infection of human coronary artery endothelial cells). The following day, the media containing recombinant retroviruses were collected, filtered through 0.45micron filter and mixed with polybrene (Sigma) up to 9ug/ml and used to feed the cells intended for infection. The next day, fresh media containing puromycin (1ug/ml) was given to the cells. After 3-4 days when all the control cells in the uninfected plates were dead, the surviving infectants were grown and used for experiments as described above.

### DNA extraction

DNA was extracted from cells using the Zymo Quick DNA mini-prep plus kit (D4069) according to the manufacturer’s instructions and DNA methylation levels were measured on Illumina 850 EPIC arrays according to the manufacturer’s instructions.

### Cellular senescence assay

Cells were trypsinised, neutralised and counted. After centrifugation at 8,000 revolutions per minute in a micro-centrifuge, cell pellet was resuspended in 200 μl reaction buffer (Cell Signaling, Senescence β-Galactosidase Staining Kit #9860) containing 10 mM FDG and 0.2% of TritonX-100. After an hour of incubation at 37^o^C, measurements of fluorescence were made with a plate reader with emission at 488 nm. The fluorescence reading is divided by the cell number to obtain the fluorescence index.

### DNA methylation data

The Illumina BeadChips (EPIC or 450K) measures bisulfite-conversion-based, single-CpG resolution DNAm levels at different CpG sites in the human genome. These data were generated by following the standard protocol of Illumina methylation assays, which quantifies methylation levels by the β value using the ratio of intensities between methylated and un-methylated alleles. Specifically, the β value is calculated from the intensity of the methylated (M corresponding to signal A) and un-methylated (U corresponding to signal B) alleles, as the ratio of fluorescent signals β = Max(M,0)/[Max(M,0)+ Max(U,0)+100]. Thus, β values range from 0 (completely un-methylated) to 1 (completely methylated). We used the "noob" normalization method, which is implemented in the "minfi" R package. Both pan-tissue clock and Skin & blood clock algorithms were previously published [26,44].

### Western blot analysis

30 μg of protein lysates were separated on 6% polyacrylamide gel and transferred onto PVDF membrane (Bio-Rad, cat. 170-4156) using semidry Trans-Blot® Turbo™ Transfer System (Bio-Rad) and high molecular weight standard protocol. Membranes were blocked in 5% TBS-T milk at room temperature for a minimum of 1 hour with gentle rocking and incubated overnight at room temperature with the following primary antibodies: hTERT (Rockland, cat. 600-401-252, 1:10 000 dilution) and GAPDH (Santa Cruz, cat. sc-25778, 1:30 000 dilution).

### Telomerase catalytic activity (TRAP) assay

Telomerase catalytic activity (TRAP) was measured using TRAPEZE® RT Telomerase Detection Kit (Millipore, cat. S7710) according to manufacturer’s instructions. Briefly, for each sample 1 million cells was resuspended in CHAPS buffer supplemented with 0.2U/μl of Superaze in inhibitor and incubated on ice for 30 min. Cellular debris were pelleted for 20 min at 12 000 g at 4 °C, supernatant aliquoted into fresh low protein binding tubes and stored at -80°C until further use; 5μl of the lysates was used for protein concentration measurement. Real-time quantitative PCR was performed using RotorGene Q. All reactions were run in triplicate using master mix provided with the kit and AccuStart II Taq DNA polymerase (Quanta Bioscience, cat. 733-2258). Equivalent of 2500 cells was used per reaction. Cycling parameters were 30 min at 30°C, then 2.0 min at 95 °C, followed by 45 cycles of 15 sec at 94 °C, 60 sec at 59 °C and 10 sec at 45 °C where fluorescence readings were taken.

Data was collected and analysed by RotorGene Q analysis software using standard curve prepared from artificial template provided with the kit. The following controls were run with the samples, positive and negative hTERT control, non-template control and heat inactivated control for each sample analysed.

## SUPPLEMENTARY MATERIAL

Supplementary Figure 1

Supplementary Figure 2

Supplementary Figure 3
