# Supplementary material for: Epigenetic ageing is distinct from senescence-mediated ageing and is not prevented by telomerase expression
Source: Aging (Albany NY). 2018 Oct 17;10(10):2800–15. doi: 10.18632/aging.101588 (PMC6224244; doi:10.18632/aging.101588)
Supplement: Supplementary Figure 1 [file aging-10-101588-s001.pdf]

Human Foreskin Fibroblasts (Donor A)

Vector p16 (55 CPD) hTERT p20 (82 CPD)

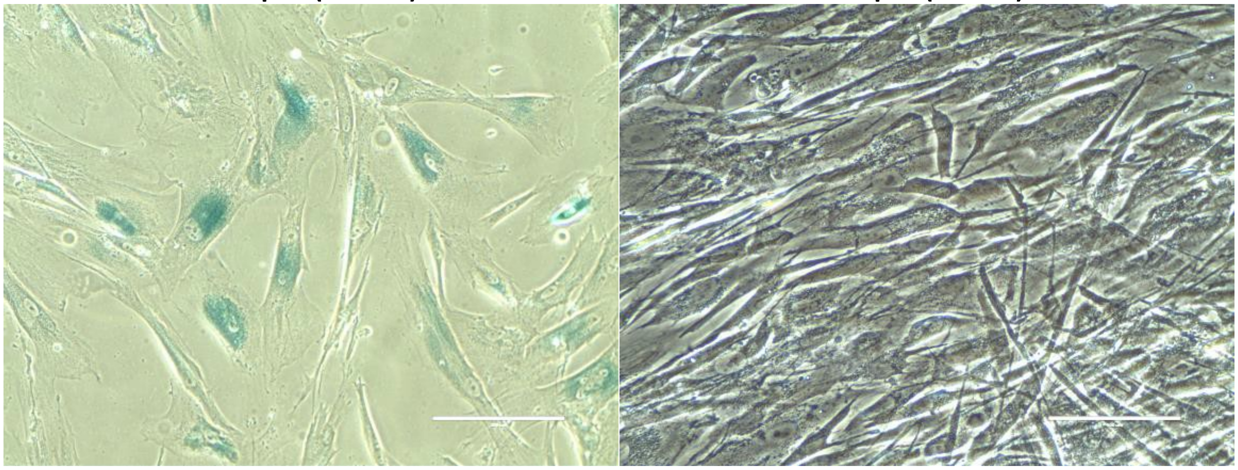

Supplementary Figure 1. Senescence-associated β-galactosidase assay on Human Foreskin Fibroblasts infected either with hTERT or empty vector at high cumulative population doubling (CPD). Scale bar measures 200µm.
