# Supplementary material for: Epigenetic ageing is distinct from senescence-mediated ageing and is not prevented by telomerase expression
Source: Aging (Albany NY). 2018 Oct 17;10(10):2800–15. doi: 10.18632/aging.101588 (PMC6224244; doi:10.18632/aging.101588)
Supplement: Supplementary Figure 2 [file aging-10-101588-s002.pdf]

Human Foreskin Fibroblasts (Donor A)

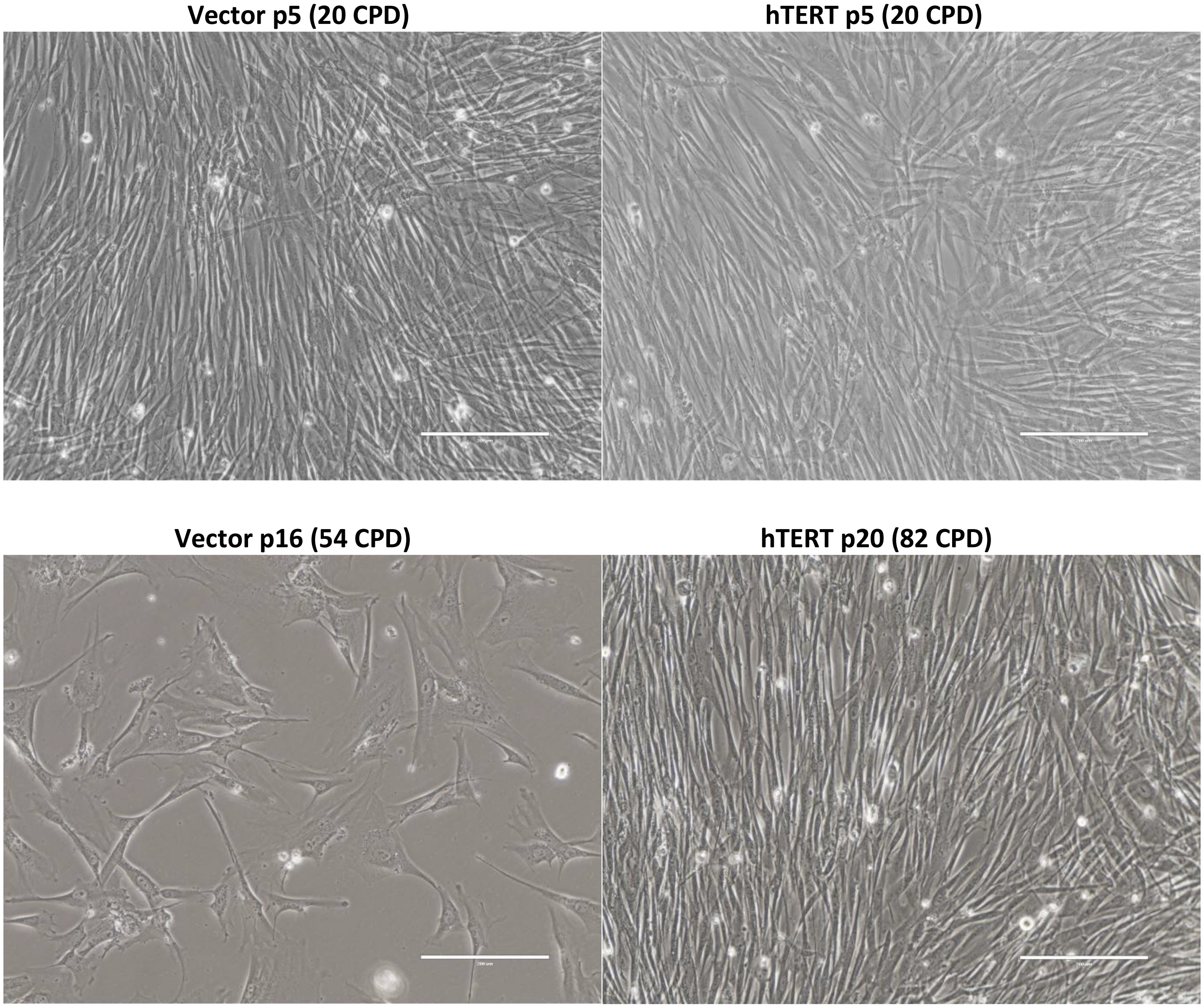

Supplementary Figure 2A. Phase contrast pictures of Human Foreskin Fibroblasts harbouring either hTERT expressing vector or empty vector at low and high cumulative population doubling (CPD). Scale bar measures 200µm.

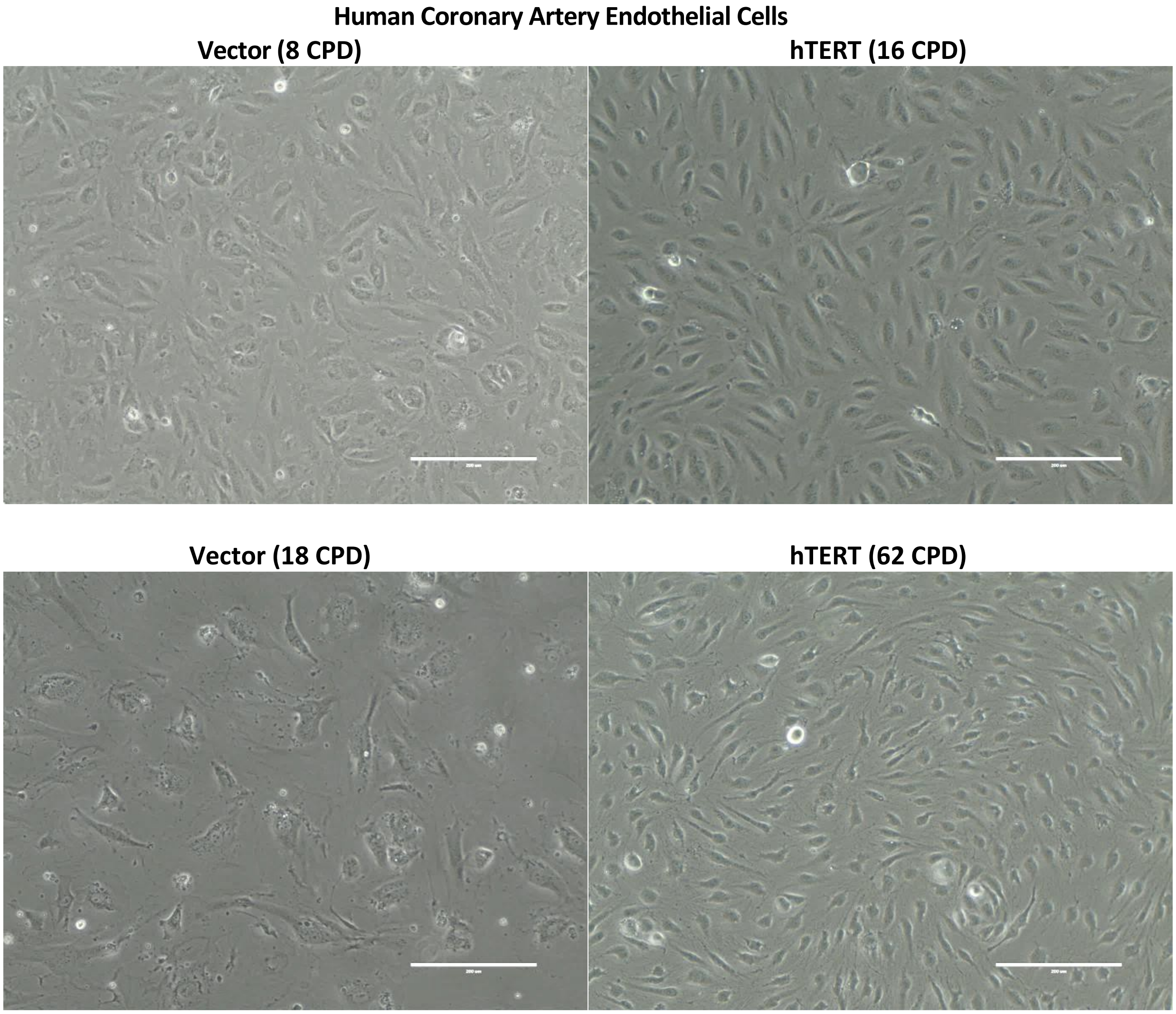

Supplementary Figure 2B. Phase contrast pictures of Human Coronary Artery Endothelial Cells harbouring either hTERT expressing vector or empty vector at low and high cumulative population doubling (CPD). Scale bar measures 200µm.
