# Supplementary material for: Epigenetic ageing is distinct from senescence-mediated ageing and is not prevented by telomerase expression
Source: Aging (Albany NY). 2018 Oct 17;10(10):2800–15. doi: 10.18632/aging.101588 (PMC6224244; doi:10.18632/aging.101588)
Supplement: Supplementary Figure 3 [file aging-10-101588-s003.pdf]

(A)

(B)

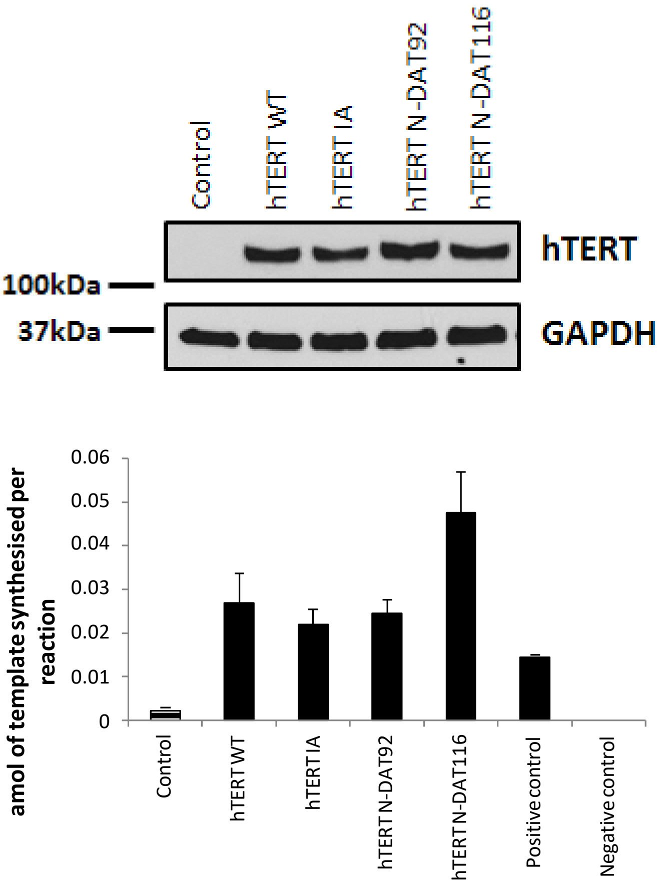

Supplementary Figure 3. (**A**) Western blot analysis of hTERT protein in human foreskin fibroblasts harbouring various hTERT constructs. (**B**) Telomerase catalytic activity (TRAP) assay on the same samples. Bars represent amount of artificial template in atto mole synthesised by telomerase in each sample.
